# Supplementary material for: Chromothripsis during telomere crisis is independent of NHEJ, and consistent with a replicative origin
Source: Genome Res. 2019 May;29(5):737–49. doi: 10.1101/gr.240705.118 (PMC6499312; doi:10.1101/gr.240705.118)
Supplement: Supplemental Material [file supp_gr.240705.118_Supplemental_file_1.zip › contigs/annotated_contigs/DB115/contig.2.DB115_length_686_mean_cov_7.43440233236.docx]

**DB115_length_686_mean_cov_7.43440233236**

```
GAGTTGGGTTTTATTTTTGGCTAAATCCCAGCGAGTCCTTG|TTATATTGGGCCCCTTGAATAAGGGTACCTTTGGGGGCCATTTGTCA
                                          >chr4:78698338-78698676 - E=2e-175 p=0e+00
CCTACTTCATACATAGCACATTTGATGAGTTTTATGTCTAGCTGGTCATCATTGATGATTGTTCAGTGATTATTTTGGCAGTTATAGCA

GGAAGATGGCACAGTATAGTTATGTGCTGCATAACAACGTTTGGATTGCCTAACAATGCATTTCTCAGAACATGTCTTTGTTAAACCAC

ACATGACTGTGCTAGTTTTGATTAGGGTGGCCATGTAACTCATCCAAATTGGACTCTTTTGAGCATGAAAAGGGATGATAATAATAATT

GCTTCAGAACAACAGATATAAACCA|TGGTGATGATGTAGATGAT|ATGTACCATGGTGATGATGTAGATTCTAATGTTAGGGAAGCTG
                                             >chr4:78696408-78696695 - E=7e-161
AAGGAGGGGTATATAGGAACTCTCTGTGTTATCTTGGTAACTTTTCTGTAAATCTAAAATTATTACAAAATTTAAAGTTATTAAAAATA

GAATATAAGTATTTTTCTTTTATGAATTGGTATAATACAGATTATATATAAAATGCCATTTTTTCCCCAGTAGGCATTCATTTGGAAGA

GTTAAACCCCACATTGTAATGTCAATAGCAAGAATTACTTGCACATTTAGCTAAAAGGTAACTGGG
```
